# DNA Methylation Dynamics Regulate the Formation of a Regenerative Wound Epithelium during Axolotl Limb Regeneration

**DOI:** 10.1371/journal.pone.0134791

**Published:** 2015-08-26

**Authors:** Cristian Aguilar, David M. Gardiner

**Affiliations:** Department of Developmental and Cell Biology, University of California Irvine, Irvine, California, United States of America; The University of Queensland, AUSTRALIA

## Abstract

The formation of a blastema during regeneration of an axolotl limb involves important changes in the behavior and function of cells at the site of injury. One of the earliest events is the formation of the wound epithelium and subsequently the apical epidermal cap, which involves *in vivo* dedifferentiation that is controlled by signaling from the nerve. We have investigated the role of epigenetic modifications to the genome as a possible mechanism for regulating changes in gene expression patterns of keratinocytes of the wound and blastema epithelium that are involved in regeneration. We report a modulation of the expression *DNMT3a*, a *de novo* DNA methyltransferase, within the first 72 hours post injury that is dependent on nerve signaling. Treatment of skin wounds on the upper forelimb with decitabine, a DNA methyltransferase inhibitor, induced changes in gene expression and cellular behavior associated with a regenerative response. Furthermore, decitabine-treated wounds were able to participate in regeneration while untreated wounds inhibited a regenerative response. Elucidation of the specific epigenetic modifications that mediate cellular dedifferentiation likely will lead to insights for initiating a regenerative response in organisms that lack this ability.

## Introduction

The remarkable ability of stem cells to be programmed to a specific fate will eventually allow many injuries and diseases to be treated more effectively, or even cured. In an effort to generate large amounts of stem cells, researchers are focused on somatic cell reprogramming (SCR) which will lead to the generation of stem cells from patients’ own somatic cells [1]. This strategy may prevent complications associated with immune rejection such as graft vs. host disease and loss of transplanted tissue. Currently, the process of SCR is highly variable and inefficient [2]. Recent evidence has suggested that the epigenetic modifications of the genome of differentiated somatic cells are a type of ‘barrier’ to the reprogramming process [3,4]. Understanding the signals that regulate epigenetic patterns associated with pluripotency during development (e.g. embryonic stem cells, ES cells) and dedifferentiation during regeneration will provide insights for the design of more efficient and reliable methods of SCR. Future SCR techniques likely will involve inducing specific patterns of epigenetic modifications mimicking that of the desired state of developmental potency [5].

Epigenetic regulation of gene expression encompasses a wide array of processes such as chromatin remodeling to increase/decrease gene accessibility, recruitment of activators or repressors of transcription, and methods of translational repression such as microRNAs [6]. The best characterized of these are post-translational modifications of histone tails and DNA methylation. Methylated cytosines in the promoter region of a gene can interfere with transcription factor binding resulting in silencing of gene expression [7,8]. In addition, recent evidence of intergenic methylation suggests a role for DNA methylation in gene expression [9]. Two main categories of DNA methylation mechanisms have been identified: maintenance methylation and *de novo* methylation. DNA methyltransferase1 (DNMT1) is responsible for maintenance methylation in which the pattern of CpG methylation is faithfully transmitted from a parent cell to a daughter cell during division [10]. The DNMT3 group of methyltransferases performs *de novo* methylation in which previously unmethylated cytosines are modified, resulting in changes in gene expression. These enzymes play key roles in embryonic development [11] and cellular differentiation [12,13,14,15], being highly expressed in undifferentiated cells (e.g. ES cells) and subsequently down regulated in cells as they differentiate. ES cells deficient in these enzymes are able to retain their pluripotent state, but are unable to differentiate unless DNMT function is restored [6,16]. On the other hand, mouse ES cells lacking all DNMTs showed increased expression of tissue specific transcription factors and signaling molecules [17], indicating that epigenetic modifications are also required to repress differentiation and maintain ES cells in an undifferentiated state.

Taken together, these findings demonstrate the important function of epigenetic modifications on the regulation of cell fate and developmental potency. During embryonic development, the epigenome changes as cells become increasing differentiated and lose developmental plasticity. The success of regenerative therapies involving induced dedifferentiation and increased plasticity likely will depend on the ability to regulate these epigenetic changes. Rather than reprogramming differentiated cells to a pluripotent state (e.g. ES-like cells), it would be more efficient to reverse the epigenetic program to the point of generating a population of lineage-specific progenitor cells (e.g. undifferentiated connective tissue progenitor cells that could regenerate cartilage, bone, ligaments and tendons). It will become important to analyze the changes to the epigenetic patterns that occur during various stages of differentiation, as is demonstrated by the changes to the methylome that occur during the differentiation of human embryonic stem cells to cardiomyocytes [18]. With this knowledge, it may not be necessary to reprogram cells to a state of complete pluripotency [19], but rather to an intermediate stage of multipotency that retains the epigenetic marks that function to stabilize the developmental state of the desired progenitor cell. By changing only the necessary epigenetic marks, the process of dedifferentiation and eventual re-differentiation of reprogrammed cells could be regulated.

Urodele amphibians such as the axolotl (*Ambystoma mexicanum)* are unique among adult vertebrates in that they are able to regenerate lost body structures perfectly, restoring previous structure and function. The success of axolotl limb regeneration is dependent on the formation of a blastema, which is structurally and functionally equivalent to a limb bud in the embryo [20]. In turn, blastema formation is dependent on signaling from a nerve that recruits undifferentiated mesenchymal cells that interact with the overlying wound epithelium [21,22]. The function of this wound epithelium (WE) is dependent on signals from the regenerating nerve that induce dedifferentiation of basal keratinocytes to form the apical epithelial cap (AEC), which is functionally equivalent to the AEC of developing amphibian limb buds and the Apical Ectodermal Ridge (AER) of developing amniote embryos. Basal keratinocytes of all three structures (blastema AEC, limb bud AEC and AER) express the transcription factor *Sp9* [22] that is involved in the regulation of FGF signaling. During regeneration, signaling from the AEC is required for dedifferentiation of cells in the limb stump (e.g. connective tissue fibroblasts) and activation of stem cells (e.g. muscle satellite cells to give rise to myoprogenitor cells), leading to the formation of a blastema [20,23]. Cells of the blastema multiply and eventually differentiate to reform the missing parts of the limb [24].

In humans, injuries to limbs and digits are common and can result in loss of the appendage as a result of the initial trauma or surgical amputation [25]. The axolotl has become the focus of research aimed at understanding the mechanisms of regeneration with the goal of being able to apply that knowledge to guide translational research to develop regenerative therapies for humans [26]. Studies from animals such as the axolotl, have demonstrated that the early nerve-dependent process leading to formation of the AEC are critical to the success of regeneration, yet little is know about the underlying cellular and molecular mechanisms regulating this process. Formation of the AEC involves re-expression of embryonic genes (e.g. *Sp9*) and re-acquisition of the ability to support blastema cell proliferation (comparable to the limb bud AEC/AER). We therefore hypothesized that nerve signaling mediates epigenetic modifications of the wound epithelium resulting in dedifferentiation of the basal keratinocytes and formation of the AEC [21,27].

To determine whether or not there are nerve-dependent epigenetic modifications of the AEC associated with blastema formation, we took advantage of the Accessory Limb Model (ALM), which is an *in vivo* gain-of-function assay for signaling that regulates dedifferentiation and blastema formation [21, 24]. In this assay, an ectopic blastema is induced on the side of the arm by making a small full-thickness skin wound and surgically deviating the brachial nerve to the wound site. Nerve-induced ectopic blastemas are equivalent to amputation-induced blastemas in terms of cellular behaviors and patterns of gene expression [24]. If a nerve is not deviated, the wound heals without forming an ectopic blastema [21]. We therefore were able to compare the differential regulation of *de novo* DNA methylation in the WE of a non-regenerating wound (no deviated nerve) and a WE that will induce formation of a blastema (deviated nerve). We report that DNMT3a expression is regulated by nerve signaling, and that experimental manipulation of DNMT3a activity can induce a regenerative response in wounds that normally would not regenerate in the axolotl. We thus have identified a source of signaling that functions to regulate epigenetic modifications associated with the initial blastema formation leading to limb regeneration.

## Results

Amputation of a salamander limb has long been the model for regeneration studies. In recent years, we have developed and optimized an alternative regeneration model, the Accessory Limb Model (ALM), which allows us to discern regeneration-specific signals that are distinct from the generalized injury signals triggered by the massive trauma of amputation [21,24]. The ALM is based on the discovery that a full-thickness skin wound on the side of the upper forelimb can be induced to form an ectopic blastema in response to signaling from a surgically deviated nerve [21,28]. This ectopic blastema is structurally and functionally equivalent to an amputation-induced blastema, and can be induced experimentally to form a well-patterned ectopic limb [21,24]. In the present study, we have used the ALM to identify and characterize changes in DNA methylation at the early stages of regeneration that are specifically associated with the response of the early wound epithelium (WE) to signaling from the nerve.

We initially screened the *Ambystoma* EST database (http://www.ambystoma.org/genome-resources/5-gene-and-est-database) for expression of genes associated with epigenetic regulation. In addition to a number of genes encoding for histone modifying enzymes, two axolotl orthologs of human DNA methyltransferases (DNMT1 and DNMT3a) were identified. Based on an initial PCR screen for changes in the level of expression of these genes during stages of regeneration, we identified DNMT3a as a candidate gene for nerve-dependent epigenetic modifications during axolotl limb regeneration.

### DNMT3a expression is induced in regenerating tissues of the axolotl limb

**Fig 1:**
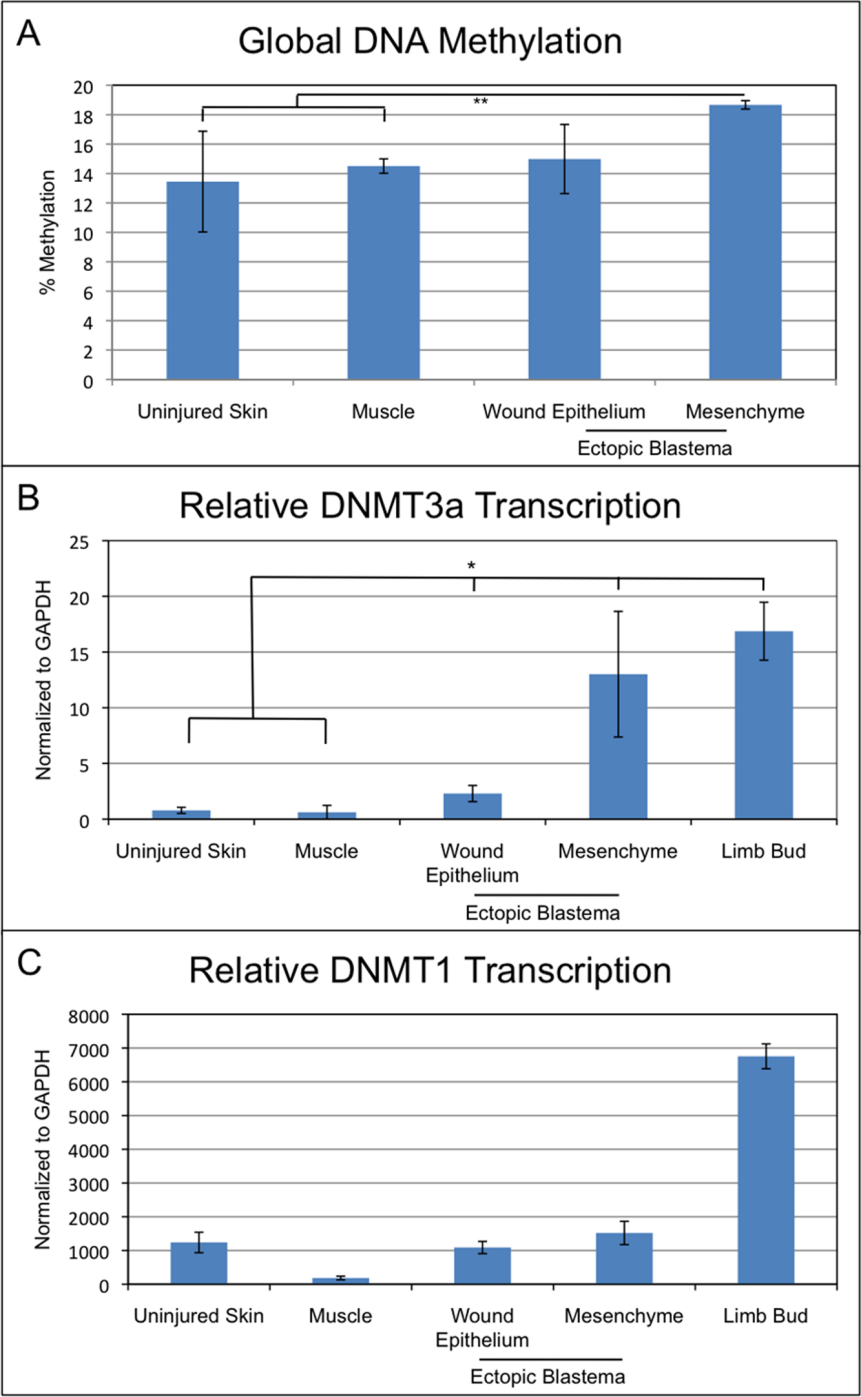
DNA methylation and methyltransferase expression in ectopic limb blastemas. (A) Total DNA methylation levels as determined through ELISA format assay. Ectopic blastema tissue samples were collected at 10 days post nerve deviation. n = 6 for each sample, totaling 24 samples. (B, C) qPCR analysis of DNMT1 and DNMT3a expression in uninjured tissues (skin and muscle), regenerating tissues (wound epithelium, ectopic blastema mesenchyme), and developing limb buds. Ectopic blastema tissue samples were collected at 10 days post nerve deviation. n = 10 for uninjured skin, muscle, wound epithelium, and mesenchyme; n = 4 for limb bud, totaling 44 samples. (* = p < 0.05; ** = p < 0.005).

As an initial characterization of epigenetic modifications associated specifically with blastema formation, we quantified changes in the global level of DNA methylation in the blastema WE and mesenchyme relative to uninjured skin and muscle tissues. Ectopic blastemas (day 10 post-surgery) that were equivalent to early bud—medium bud blastemas that form on an amputated limb were collected, and global DNA methylation was analyzed separately for blastema epithelial and mesenchymal cells (Fig 1A). The 10 day time point was used in order to collect sufficient tissue from the blastema mesenchyme for analysis and comparison with regenerative and non-regenerative tissues. Although the level of DNA methylation of the blastema WE was increased relative to the uninjured skin, this difference was not statistically significant. The level of global DNA methylation of the blastema mesenchymal cells was significantly increased compared to stump muscle and uninjured skin. Since approximately 50% of the early blastema mesenchymal cells are derived from dermal fibroblasts of the uninjured skin [29], the approximately 4% increase in the level of methylated cytosines associated with the transition from dermal cell to blastema cell suggests a regeneration-specific role for DNA methyltransferases in blastema formation.

Much of the increase in global DNA methylation of blastema cells appeared to be a consequence of increased levels of DNMT3a expression. Expression of both DNMT1 and DNT3a was increased in ectopic blastemas, but only DNMT3a expression appeared to be regeneration-specific (Fig 1B and 1C). The expression of DNMT3a, a *de novo* DNA methyltransferase, was significantly higher in both the WE and mesenchyme of early/medium-bud ectopic blastemas relative to the uninjured control tissues (Fig 1B). Furthermore, blastema mesenchyme cells expressed DNMT3a at levels that were comparable to developing limb bud cells. Although DNMT1, a maintenance methyltransferase, was expressed at a higher level relative to stump muscle tissues, its level of expression was not significantly different between ectopic blastema tissues and uninjured, full-thickness skin (Fig 1C). Since expression of DNMT1 is associated with cell division, an increased level of expression would be expected in the skin since it is a continuously proliferating tissue. The highest levels of DNMT1 expression were measured in developing limb bud cells that have a high rate of proliferation. Taken together, these data suggested that the upregulation of DNMT3a expression could account for the increase in global methylation levels of cells of the blastema as compared to the progenitor cells in the uninjured skin. Thus, we hypothesized that *de novo* methylation leading to new cytosine methylation sites within the genome of cells of a regenerating limb is linked to dedifferentiation and blastema formation.

### Expression of DNMT3a is modulated by nerve signaling

**Fig 2:**
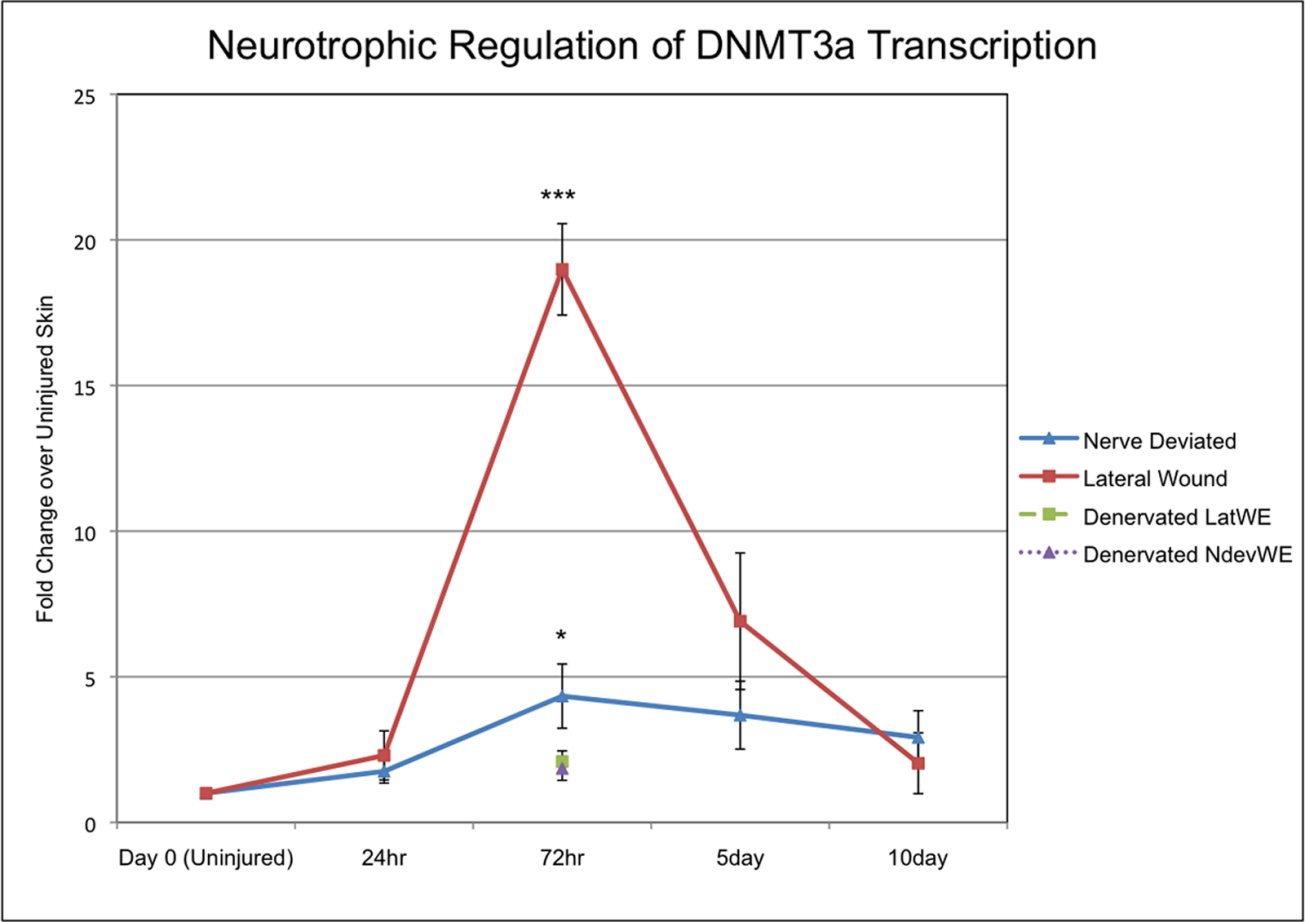
DNMT3a expression is modulated by signaling from nerves. DNMT3a expression (qPCR) in the epithelium of wounds created on the arm of axolotls that either healed without forming a blastema (lateral wound; n = 8 for day 0, 24hr, 72hr, 5 days. n = 10 for 10 days) or received a surgically deviated nerve to form an ectopic blastema (nerve deviated; n = 8 for day 0, 24hr, 72hr, 5 days. n = 10 for 10 days). In addition the nerve supply to the limb was severed proximally (denervated) prior to making wounds either with (NdevWE; n = 4) or without (LatWE; n = 4) surgically deviating a nerve distally. (* = p < 0.05; *** = p < 0.0005).

The success of regeneration is dependent on the progression through multiple steps, which in turn are dependent on signaling from nerves [21]. One of the earliest steps requires nerve signaling in order for the keratinocytes of the WE to dedifferentiate and acquire the signaling properties of the AEC that is required for blastema formation [22,27]. To determine whether changes in *de novo* DNA methylation are associated with the transition from an early WE to an AEC, we analyzed the expression of DNMT3a in keratinocytes of the WE/AEC during the first 10 days of ectopic blastema formation. Expression of DNMT3a in a WE with a deviated nerve increased significantly (about 4-fold) compared to expression in the uninjured skin (Fig 2). Expression was highest at 72 hours after initial wounding and declined slightly over the next 7 days. Surprisingly, DNMT3a expression increased dramatically in a WE that did not have a surgically deviated nerve (19-fold compared to uninjured skin; more than 4-fold greater than in a WE with a deviated nerve). This difference between wounds that did or did not have a deviated nerve was transient such that there was no difference in the level of DNMT3a expression at either 24 hours or 10 days post injury. Therefore wounds without a deviated nerve that regenerated the skin, but did not form ectopic blastemas, formed a WE that expressed DNMT3a transiently at high levels compared to wounds with a WE that was induced by nerve signaling to form an AEC and an ectopic blastema.

Wounds without a deviated nerve were created on limbs that were innervated, and consequently there was a low level of innervation of the wound even though a nerve was not surgically deviated [22]. In order to eliminate the influence of nerve signaling entirely, we surgically denervated limbs proximally at the brachial plexus, and then made skin wounds that did or did not have a surgically deviated nerve distally. For both treatments, expression of DNMT3a was not detected at either early (24 hours post-wounding) or late (5 or 10 days post-wounding) time points. There was a small, transient increase (2-fold) increase at the 72 hour time point (Fig 2). Since a nerve that has been denervated proximal and deviated distally does not rescue induced DNMT3a expression, we assume that the signaling that regulated DNMT3a expression was associated with viable nerves and not other cells associated with the nerve (e.g. Schwann cells). Both too high and too low a level of DNMT3a expression was associated with the failure to form a blastema, which is consistent with an hypothesis that AEC function is dependent on the quantitative regulation of DNMT3a expression by nerve signaling.

### Inhibition of DNMT activity induces *Sp9* expression

Increased nerve signaling from a deviated nerve is required for AEC formation [22,27] and is associated with downregulation of DNMT3a expression (this study). We therefore tested whether nerve-independent downregulation of DNMT3a activity would be sufficient to induce AEC formation. To do this, we used 5-aza-2’-deoxycytidine, also known as decitabine (abbreviated here as Dec), to inhibit DNMT activity through its inability to receive the addition of a methyl group to the carbon at the 5 position of cytidine when incorporated into DNA during the S phase of the cell cycle. 2’-deoxycytidine (abbreviated here as 2’dC), the standard DNA nucleotide, was used as a control treatment. Incorporation of 2’-dC into the DNA of dividing cells would not affect DNA methyltransferase activity as it is the normal substrate of these enzymes. Sol-gel beads (1mm in diameter) that contained either Dec or 2’-dC were synthesized and grafted under the wound epithelium of lateral wounds 24 hours after the initial surgery to create the full-thickness skin wounds.

**Fig 3:**
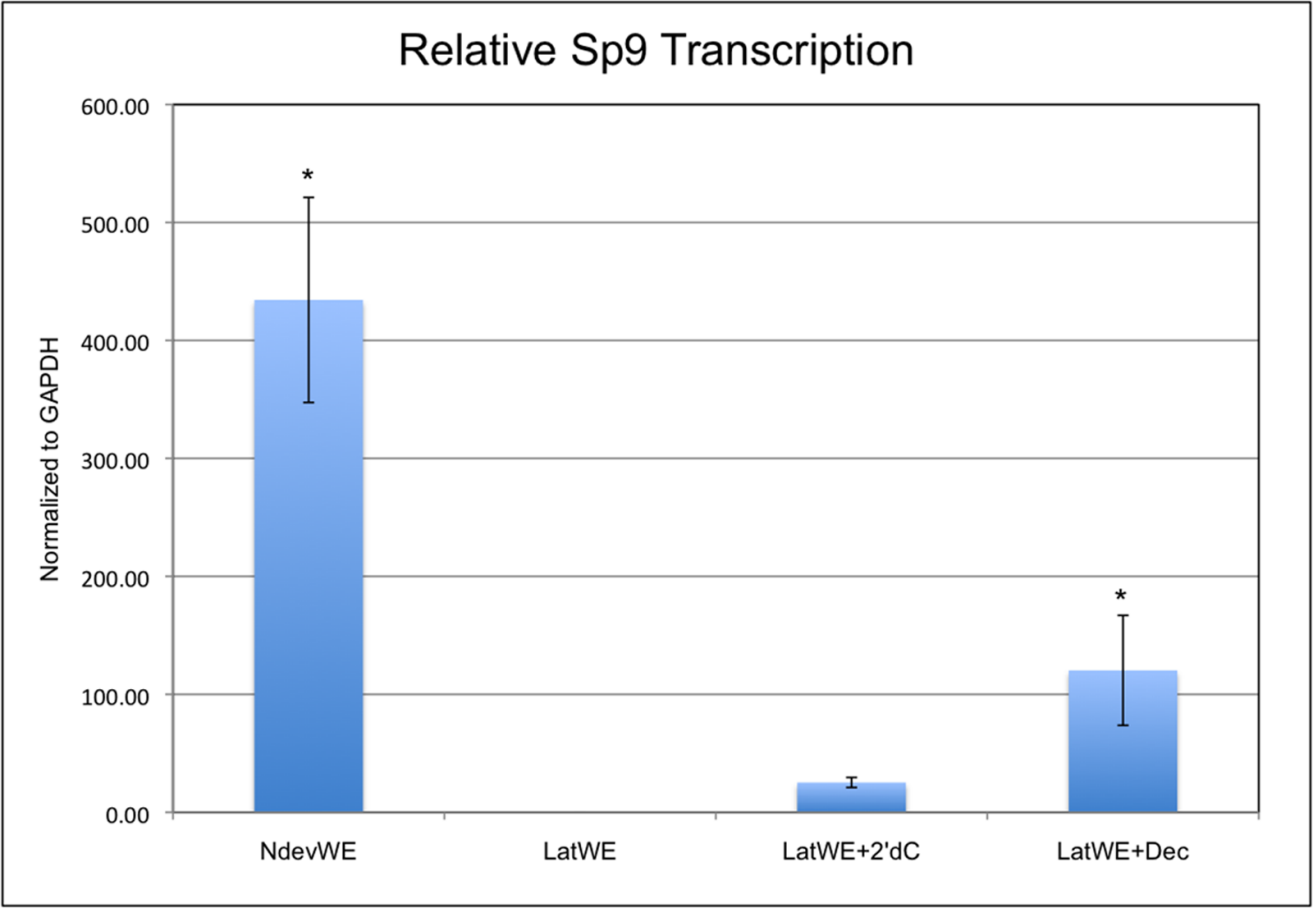
Inhibition of DNMT activity induces expression of the WE/AEC marker gene, *Sp9*. *Sp9* expression (qPCR) in the epithelium of wounds created on the arm of axolotls that healed without forming a blastema (lateral wound epithelium, LatWE; n = 5); received a surgically deviated nerve to induce formation of an ectopic blastema (nerve deviated wound epithelium, NdevWE; n = 7); received an implanted bead containing 2’deoxycytidine without a deviated nerve (LatWE + 2’dC; n = 5); or received an implanted bead containing decitabine without a deviated nerve (LatWE + decitabine; n = 7). (* = p < 0.05).

To assay for a regenerative response associated with the inhibition of DNMT activity, we quantified the expression of *Sp9*, a marker gene for the basal keratinocytes of the AEC [22,27]. *Sp9* is a zinc finger transcription factor that is expressed in the AER of developing limb buds and is re-expressed in the AEC during limb regeneration. In response to signaling from a surgically deviated nerve, *Sp9* expression is induced throughout the WE within 24 hours after wounding, and becomes localized to the basal keratinocytes of the AEC 72 hours after the initial injury. In contrast, *Sp9* expression is not detected in wounds without a deviated nerve at 72 hours after injury [22]. We therefore grafted beads with Dec or 2’-dC into wounds that did not have a surgically deviated nerve. Wounds that received a grafted Dec bead re-expressed *Sp9* in the absence of a deviated nerve (Fig 3) at a statistically higher level than wounds that received a control bead (2’-dC). Although the level of *Sp9* expression was lower than for nerve-deviated wounds, the disparity was not statistically different, and presumably was a consequence of the need to have decitabine incorporated into the replicating DNA of the target cells of the WE in order to inhibit DNA methylation. As reported previously, *Sp9* expression was not detected in wounds without either a grafted bead or a deviated nerve; however, there was a low level increase in *Sp9* expression in control wounds receiving 2’-dC beads. This level of expression was significantly lower than both the nerve-deviated and the decitabine-treated wounds, and may have been a consequence of reinjuring the wound at 24 hours when the bead was grafted. Although decitabine treatment induced expression of a gene associated with formation of WE/AEC, it did not induce formation of an ectopic blastema. Therefore it appears that downregulation of DNMT activity is not sufficient to induce blastema formation, and that additional signaling pathways are involved in the early stages of regeneration.

### Inhibition of DNMT activity delays reformation of the basal lamina of the WE

Skin wounds without a deviated nerve, as well as the amputation wounds of denervated limbs, reform a basal lamina within a few days of injury. In contrast, the basal lamina underlying the AEC of both nerve-induced ectopic blastemas and amputation-induced blastemas does not reform until the end of regeneration [21,22,30]. Taken together, these observations have led to the hypothesis that the presence of a basal lamina inhibits signaling between the AEC and blastema mesenchyme, and therefore blastema formation and outgrowth only can occur if reformation of the basal lamina is inhibited [30]. This regulation of basal lamina regeneration is hypothesized to be mediated by interactions between the nerve and the basal keratinocytes of the AEC [27].

**Fig 4:**
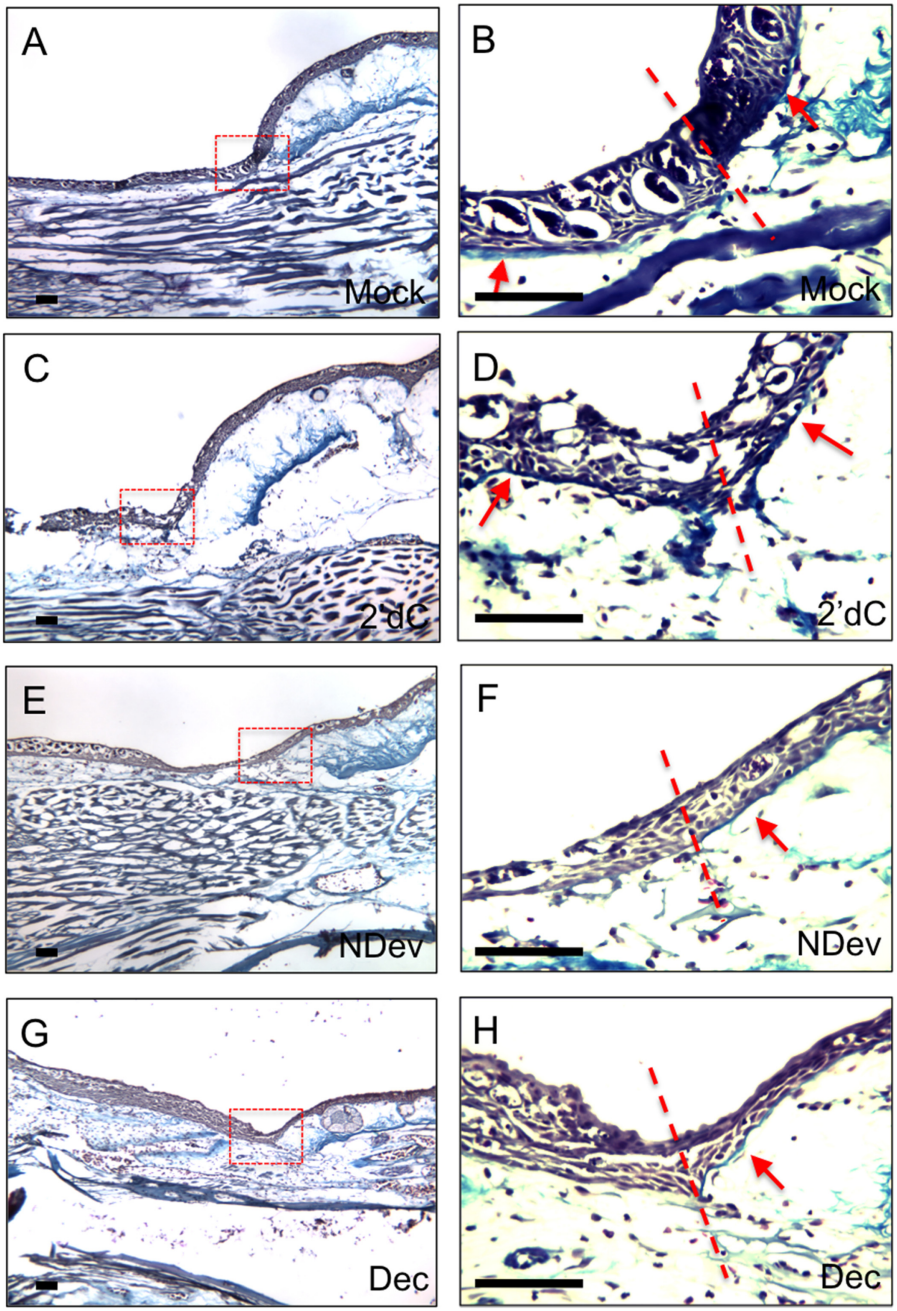
Inhibition of DNMT activity inhibits reformation of the basal lamina. Trichrome staining of wounds six days post-surgery. Wounds were either untreated (A, B; mock; n = 4); received an implanted bead containing 2’deoxycytidine without a deviated nerve (C, D; 2’dC; n = 8); received a surgically deviated nerve to induce formation of an ectopic blastema (E, F; NDev; n = 4); or received an implanted bead containing decitabine without a deviated nerve (G, H; Dec; n = 8). Images in (B, D, F and H are higher magnifications of the boxed areas in (A, D, E, and G correspondingly). In order to increase the visibility of the basal lamina (stained blute) in the original images, the high magnification images (panels B,D,F,H) have been color adjusted by placing the mid-tone colors on the blue end of the spectrum and highlights on the yellow end of the spectrum. All four panels were adjusted as one image, and thus have been treated equally. The lower magnification images were not adjusted. Dotted lines indicate the transition between the uninjured skin (right) and the wound (left). Arrows indicate the region beneath the epidermis/WE where the basal lamina structure can be detected. Scale bars = 200 microns.

**Fig 5:**
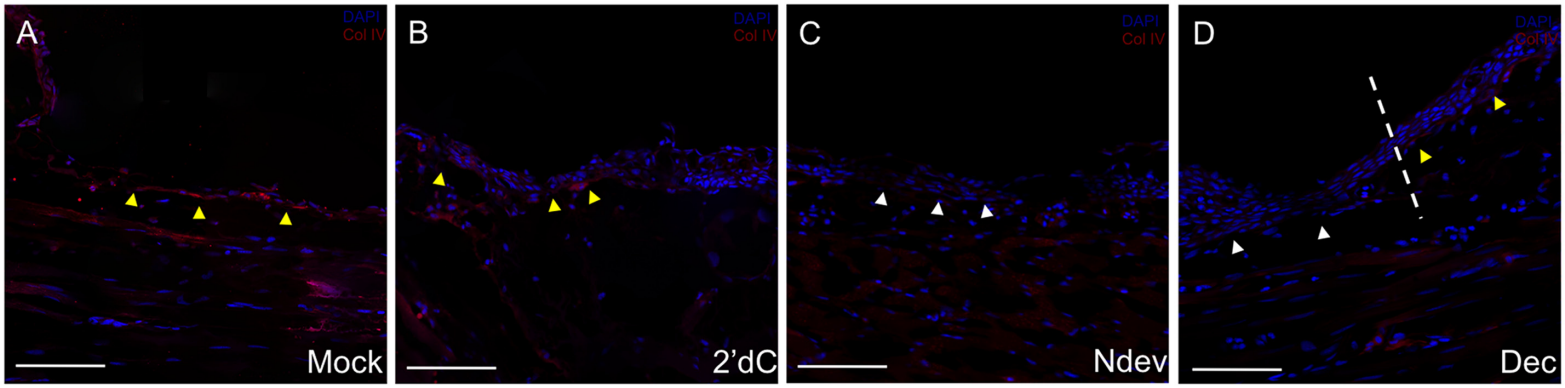
Inhibition of Collagen IV expression in decitabine-treated wound epithelia. Immunohistochemistry staining for Collagen IV, a marker for the basal lamina, in wounds six days post-surgery. Wounds were either untreated (A, mock); received an implanted bead containing 2’deoxycytidine without a deviated nerve (B, 2’dC); received a surgically deviated nerve to induce formation of an ectopic blastema (C, NDev); or received an implanted bead containing decitabine without a deviated nerve (D, Dec). White arrowheads indicate areas within the wound that are negative for ColIV staining; yellow arrowheads indicate areas that are positive for Col IV staining. Dotted line in (D) indicates the transition between the uninjured skin (right) and the wound (left). Scale bars = 200 microns.

To assay for a regenerative response associated with the inhibition of DNMT activity, we examined wounds that had been treated with decitabine for the presence of a basal lamina. We used trichrome staining (blue) to visualize the thin collagen layer beneath the basal keratinocytes of the WE as it reformed the basal lamina. Limbs with wounds were collected 6 days after the initial surgery to create a wound (5 days after bead grafting), sectioned, and stained for the presence of the basal lamina. The presence or absence of the basal lamina underlying the WE is best visualized at the border of the wounds where comparison can be made with the presence of the basal lamina and dense collagen fibers of the dermis beneath the uninjured skin. As reported previously [21], wounds without a deviated nerve or a grafted bead reformed a basal lamina (Fig 4A and 4B); whereas, nerve-deviated wounds did not reform the basal lamina underneath the WE/AEC (Fig 4E and 4F). Control wounds that received 2’-deoxycytidine bead grafts reformed the basal lamina (Fig 4C and 4D), and appeared similar to lateral wounds without a deviated nerve (Fig 4A and 4B). In contrast, wounds that received decitabine beads did not reform the basal lamina (Fig 4G and 4H), and appeared similar to nerve-deviated wounds (Fig 4E and 4F). Additionally, immunohistochemistry staining for collagen IV confirmed the delay of basal lamina formation in regenerating wounds and lateral wounds treated with decitabine (Fig 5). Within mock grafted wounds and control wounds receiving a 2’-deoxycytidine bead, collagen IV staining was detected beneath the wound epithelium (Fig 5A and 5B). Wounds that received a deviated nerve to initiate the regenerative response had not reformed the basal lamina at the 6 day time point (Fig 5C). Lateral wounds that would normally reform the basal lamina within the 6 day experimental window exhibited a delay after decitabine treatment, similar to nerve deviated wounds (Fig 5D). Although reformation of the basal lamina was delayed, an ectopic blastema did not form, indicating that downregulation of DNMT activity is associated with but not sufficient for blastema formation. At this point we cannot determine whether the observed delay in basal lamina reformation is a consequence of the downregulation of DNMT activity in the WE/AEC, the underlying mesenchymal cells of the stump, or both.

### Decitabine treated wound epithelia participate in blastema formation

**Fig 6:**
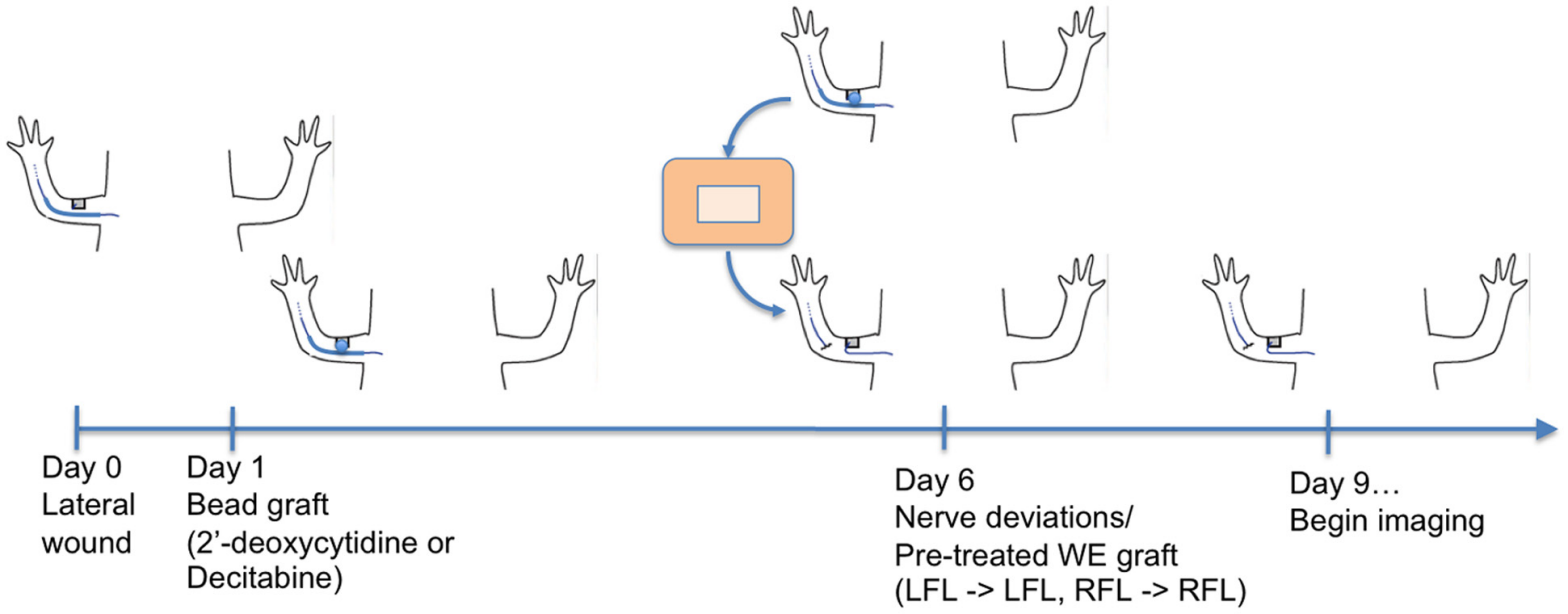
Experimental design to test whether decitabine-treated wound epithelia can participate in blastema formation. Cartoon illustrating the sequence of surgical procedures for making the wound, implanting beads, and grafting the treated and control wound epithelia to a new wound with a deviated nerve.

Re-expression of *Sp9* and the delay of basal lamina formation are both hallmarks of the early stages of regeneration, and both appear to be mediated by interactions between the nerve and the WE. In order to test the hypothesis that the regulation of DNMT activity by the nerve in turn regulates the pro-regenerative activity of the WE, we assayed for the ability of a grafted WE/AEC to participate in blastema formation. Full-thickness skin wounds were created on the anterior side of the upper arm, and either 2’-dC beads or Dec beads were grafted 24 hours later (Fig 6). Six days after initial injury (five days after bead grafting) the wounds were collected such that there was a border of previously uninjured skin surrounding the original wound, and were grafted onto a new host wound along with a surgically deviated nerve. The graft was positioned such that the WE/AEC of the graft was localized centrally above the deviated nerve. In the absence of a graft, the new host wound would normally heal and form a functional WE/AEC and an ectopic blastema, but the grafted WE/AEC with a border of uninjured skin prevented the host wound from forming a new WE/AEC. We therefore tested whether or not the grafted WE/AEC could respond to the deviated nerve and function in the formation of an ectopic blastema.

**Fig 7:**
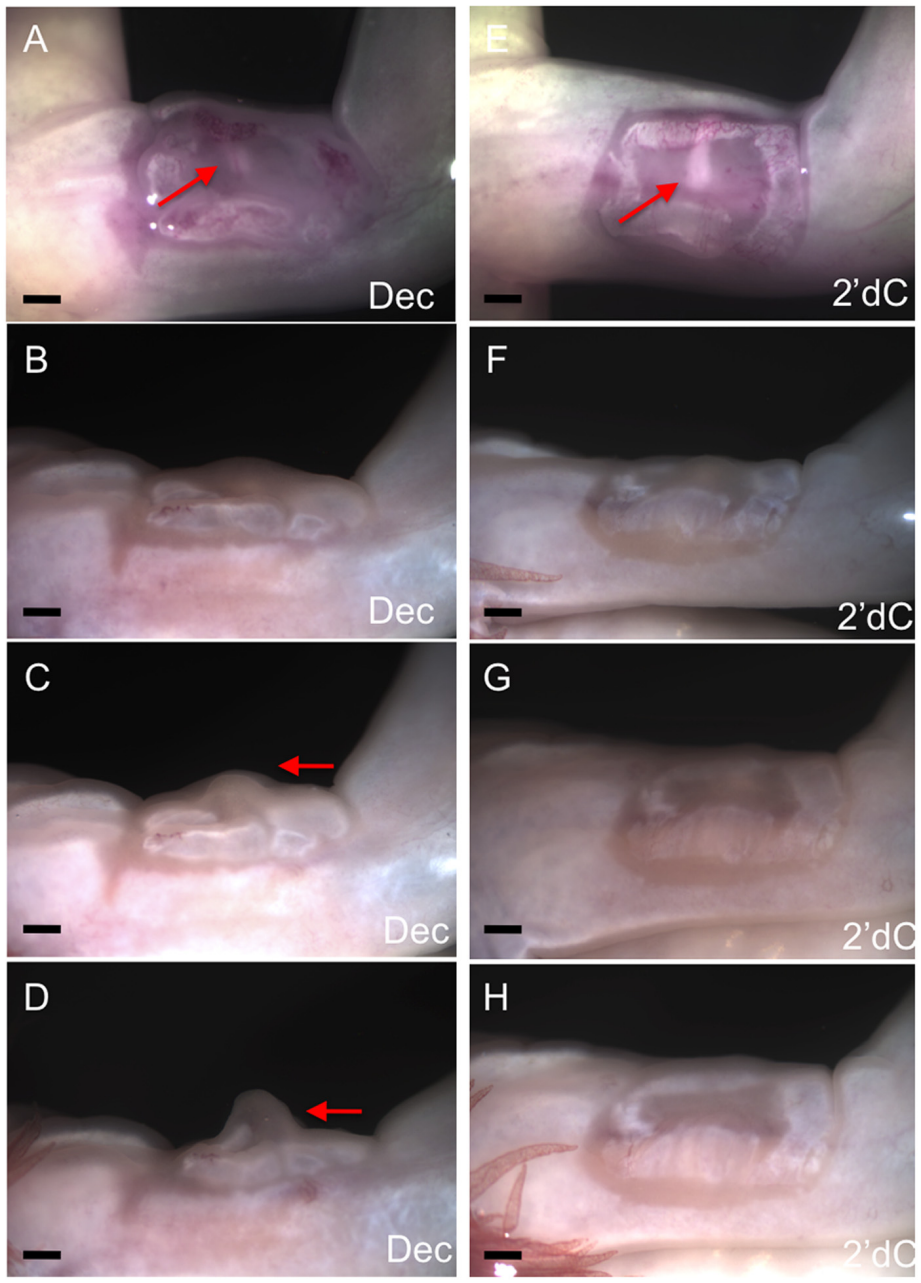
Decitabine treated wound epithelia can participate in blastema formation. Participation of a decitabine-treated WE in blastema formation as observed at day 3 (A), day 7 (B), day 11 (C) and day 16 (D) after grafting; n = 6. Lack of formation of an ectopic blastema when a 2’deoxycytidine-treated WE was grafted as observed at day 3 (E), day 7 (F), day 11 (G) and day 16 (H) after grafting; n = 8. Arrows indicate the position of the deviated nerve (A, E) and the formation of an ectopic blastema (C, D). Scale bars = 1mm.

Although control (2’-dC treated) and decitabine-treated wounds appeared similar six days after the initial wounding, most of the decitabine-treated wounds participated in blastema formation when grafted to a nerve-deviated host wound (4 of 6); whereas, control wounds did not (Fig 7 and Table 1). In some host wounds, a blastema formed in the small region between the peripheral margin of the grafted WE/AEC and host wound border (a “secondary blastema’). This region surrounding the grafted tissue can be visualized in the images of Fig 7 as a thin border that becomes re-epithelialized following the introduction of the graft. In one limb, there was a tear in the grafted WE/AEC of a control wound that re-healed and formed a new WE/AEC that participated in blastema formation (Table 1). The ectopic blastemas formed with decitabine treated WE/AEC developed to the medium bud stage before regressing, which is what occurs with nerve-induced blastemas [21]. The overall time-course of blastema formation and regression was similar to that of ectopic blastemas as reported previously [21]. Thus down regulation of DNMT activity was not sufficient to induce ectopic blastema formation, but was sufficient to maintain the WE/AEC in a regeneration-competent state (consistent with the induced expression of *Sp9*) so as to be able to participate in ectopic blastema formation when grafted. At this point we cannot determine whether the keratinocytes of the WE/AEC are the direct targets of DNMT activity; nevertheless, the experimental downregulation of DNMT activity allows the WE/AEC to respond to the signals that control the early stages of regeneration leading to blastema formation.

**Table 1:** Ability of decitabine-treated wound epithelia to participate in blastema formation.

Treatment	Total	1° Blastema	No Blastema	2° Blastema
Decitabine	6	4 (67%)	2 (33%)*	0 (0%)
2’-deoxycytidine (control)	8	1** (12%)	5 (63%)	2 (25%)

* The presence of a decitabine bead could not be confirmed 24 hour after grafting in two additional wounds that did not form an ectopic blastema (a total of 8 limbs were grafted), and those data were not included.

** A tear in the WE/AEC of this wound that re-healed and formed a new WE/AEC was observed 24 hours after grafting.

## Discussion

In this paper we provide evidence that *de novo* DNA methylation is regulated in part by nerve signals during the early stages of limb regeneration. Specifically, expression of DNMT3a in keratinocytes of the WE was down regulated in response to increased nerve signaling (the presence of a surgically deviated nerve), and was associated with the transition of the WE to the AEC of the early blastema. When DNMT activity was inhibited experimentally by implanting beads that release decitabine, re-expression of *Sp9* was induced, and reformation of the basal lamina was delayed. Both of these phenomena are hallmarks of the early stages of regeneration, and both appear to be mediated by interactions between the nerve and the WE/AEC [22,27]. Our discovery that decitabine-treated WE/AEC can participate in early blastema formation is consistent with the hypothesis that nerve signals regulate *de novo* DNA methylation in keratinocytes of the WE leading to formation of the functional AEC required for blastema formation.

The formation of a functional WE/AEC during the early stages of regeneration is required for progression to the subsequent steps of the regeneration cascade [21,31]. The lack of this specialized structure or the surgical replacement with full thickness skin prevents the regenerative response, even in the presence of nerve signaling [32]. The WE/AEC is a relatively simple structure composed of a few layers of keratinocytes along with interspersed Leydig cells. Of these cells, the basal layer of keratinocytes that are adjacent to the underlying mesenchymal cells appear to function in promoting blastema formation and outgrowth [22,27]. We therefore hypothesize that nerve signals target the basal keratinocytes so as to regulate *de novo* DNA methylation associated with dedifferentiation and blastema formation. Given the ability of this population of cells to respond to nerve signals and to serve subsequently as a signal center for proliferation of the underlying blastema mesenchymal cells identifies them as important for further research.

The variable response of DNMT3a expression to different amounts of nerve signaling could be related functionally to the well-documented phenomenon of a neurotrophic threshold for regeneration [33]. Regeneration is dependent on a threshold number of nerves being present in the stump, and regeneration fails to occur below that threshold [33,34]. The ALM demonstrates this phenomenon in that denervated limbs fail to regenerate (no nerves), wounds without a deviated nerve do not make a blastema (below the threshold), and wounds with a deviated nerve make an ectopic blastema (above the threshold). The variable levels of DNMT3a expression mirrored this regenerative response to variation in the level of innervation. In the absence of nerves (denervated limbs), DNMT3a was not expressed and regeneration failed to occur. In a normal skin wound, there was innervation by sensory nerves [22], DNMT3a was expressed at high levels, and no blastema formed. In response to a deviated nerve (increased levels of nerve signaling), DNMT3a expression increased to moderate levels and a blastema formed. Thus DNMT3a expression appeared to be required for blastema formation, but the increased level of expressed must be modulated by endogenous signals from nerves. Because the experimental inhibition of DNA methylation by decitabine can induce some of the responses induced by nerve signals, this modulation could be quantitative (just enough and not too much *de novo* methylation). Since decitabine treatment did not induce nerve-independent ectopic blastema formation, there must be additional signals from the nerve that are required for blastema formation. Therefore DNMT3a modulation also could be qualitative such that nerve signals target DNMT3a to specific regions of DNA by controlling the expression of cofactors that convey specificity to DNMT3a activity. The re-expression of the transcription factor *Sp9* after DNMT inhibition may be an example of one such targeting. It is possible that DNA methylation occurs directly at the promoter of *Sp9* resulting in its silencing in non-regenerating wounds, but that the repression of DNMT3a expression in response to nerve signaling allows *Sp9* to be re-expressed. It is also possible that changes in DNA methylation indirectly result in *Sp9* re-expression by allowing for the expression of transcription factors necessary to induce *Sp9* transcription. Likewise, genes controlling the synthesis and deposition of basal lamina components are likely under the (direct or indirect) control of DNA methylation. Ultimately, the changes in the methylation states of specific nucleotides will be important to investigate. While changes in total amounts of DNA methylation inform us of a role in regeneration, there may be smaller losses and gains in DNA methylation states that are summated as no net difference.

The role of DNA methylation in the regulation of limb regeneration has not been investigated previously. In fact, it would not have been possible to discover that the level of DNMT3a expression is modulated by changes in the level of nerve signaling in the amputation model for limb regeneration. Although further studies of the quantitative dose-response relationship between nerve signaling and regenerative responses (e.g. *de novo* DNA methylation) will require the development of *in vitro* models for blastema formation and growth (work in progress), the ALM has allowed us to discover differences in the regenerative response to no nerve signaling (denervation), low nerve signaling (wounds without deviated nerve) and high nerve signaling (deviated nerve). There obviously are differences between blastema formation in response to limb amputation in which there is extensive tissue damage, and ectopic blastema formation in which non-specific damage is minimized and pro-regenerative signals (WE and nerve) are provided. Nevertheless, the induced blastemas from these two models for limb regeneration are equivalent in terms of cellular behaviors and patterns of gene expression, and both can form limbs with the same pattern [24]. Thus both models presumably use the same conserved mechanisms as used for limb development in the embryo [20], and discoveries from experiments in either model are relevant for understanding how to induce regeneration in other organisms (e.g. humans).

DNA methylation has long been recognized as a key regulatory mechanism of gene expression during development, cancer progression, and the control of differentiation states in stem cells [12,13,14,15,35,36]. Therefore, it is not surprising that DNA methylation also functions to control cell behavior and fate decisions during regeneration as indicated by the results from the present study. The ALM has allowed us to manipulate levels of nerve signaling experimentally in order to identify DNMT3a expression and DNA methylation as early critical events in the regeneration cascade. The cells of the WE/AEC respond to nerve signaling between 24 hours and 72 hours, and this well-defined window of time provides an experimental model to identify upstream regulation of functionally important epigenetic modifications. Future identification of the subset of genes that are directly affected by changes in their methylation states during blastema formation will provide insights into future therapies aimed at enhancing the human body’s inherent, although limited, regenerative responses.

## Materials and Methods

### Ethics Statement

This study was carried out in accordance with the recommendations in the Guide for Care and Use of Laboratory Animals of the National Institutes of Health. The experimental work was conducted in accordance with procedures specifically approved by the Institutional Animal Care and Use Committee of the University of California Irvine (IACUC # 2007–2705). Animals were anesthetized prior to all procedures in a 0.1% solution of MS222 (Ethyl 3-aminobenzoate methanesulfonate salt).

### Animals

Experiments were performed on white and wild-type axolotls (*Ambystoma mexicanum*) measuring 12–15 cm snout to tail tip that were spawn at the University of California Irvine or at the Ambystoma Genetic Stock Center at the University of Kentucky. The animals were maintained in 40% Holtfreter’s solution and were anesthetized prior to all procedures in a 0.1% solution of MS222 (Ethyl 3-aminobenzoate methanesulfonate salt, Sigma), pH 7.4.

### Surgical Procedures

The technique for inducing a regenerative response from wounds on the side of the limb has been described in detail previously [21,24]. Briefly, full-thickness skin wounds on the anterior side of the limb were created by surgically removing a square of skin (3–4 mm on a side) from the anterior side of the stylopod (region of the humerus/femur), making sure that the underlying muscle was not damaged. The brachial nerve then was deviated surgically beneath the skin to bring the cut end of the nerve to the center of the skin wound. Microcarrier beads were implanted into the wound site 24 hours after the initial surgery by making a small incision through the uninjured skin proximal to the wound site. Forceps were used to create a tunnel under the wound epithelium, and a bead was inserted into the center of the wound site. Tissue samples for analysis of *Sp9* transcription were collected 72 hours after the initial surgery to create the wound (48 hours after bead implantation). Samples for histological analysis were collected 6 days post wounding. For the experiment testing whether or not the wound epithelium was permissive for regeneration, wounds were created and beads were implanted into wounds 24 hours after the initial surgery. These wounds were allowed to heal for 6 days, after which the graft was collected by surgically removing a piece of full-thickness skin that contained the original would in the center. The graft was placed into a host wound with a deviated nerve such that the severed end of the nerve was localized beneath the wound epithelium from the original wound. The full-thickness skin surrounding the original wound epithelium healed into place adjacent to the skin of the host wound site.

### Bead Synthesis

Sol-gel beads were synthesized as described in [37]. Briefly, a solution of tetramethyl orthosilicate (TMOS) and methanol was mixed with either Decitabine or 2’-deoxycytidine solutions to yield a final concentration of 1μM. This solution was then pipetted in 1 μL volume drops onto a sheet of parafilm. The drops flattened when first pipetted such that they formed a hemispherical bead with a diameter of 1 mm. The beads were allowed to harden for 3 days at room temperature in a fume hood, and dried at 37°C overnight prior to grafting.

### Histology

Samples were collected 6 days after the initial surgery to create the wound, fixed in 4% PFA, and embedded in OCT for cryosectioning. Sections were stained using Mallory’s triple stain for collagen and counterstained with Hematoxylin.

### Quantification of global DNA methylation

DNA was isolated from tissues using the Invitrogen Trizol reagent. Methylation levels were assayed using the Epigentek Methylamp Global DNA Methylation Quantification Ultra Kit following the manufacturer’s protocol.

### Analysis of gene expression by qPCR

Blastema tissue samples (wound epithelium and mesenchyme) were collected from ectopic blastemas that developed on the anterior side of the arm 10 days after creating wounds with deviated nerves (surgical details described above). Samples were collected by making surgical incisions through the full thickness skin surrounding the ectopic blastema. The excised skin, including the wound epithelium was lifted from the blastema, and the border of mature skin was trimmed away from the wound epithelium. The mesenchymal portion of the blastema was then collected by surgically excising it from the side of the arm. Samples were placed in Trizol reagent and homogenized using a 20-gauge needle and syringe. RNA was isolated after chloroform induced phase separation, and purified using the Machery-Nagel NucleoSpin RNA XS kit. cDNA synthesis was performed with the Roche Transcriptor First Strand cDNA Synthesis kit. Roche SYBR green reagent was used for PCR quantification.

### Statistical analysis

Statistical significance was determined for all quantitative analyses by one-tailed students t-test, with a maximum p-value of 0.05 unless otherwise stated. Error bars in all figures represent the standard error of the mean.

### Immunohistochemistry

Slides were rehydrated and antigen retrieval was performed with proteinase K treatment (DAKO) for 4 minutes. After washing in TBST, slides were blocked for streptavidin and biotin (Vector labs) and incubated with primary antibody (1:500 collagen type IV, Rockland, 600-406-106) overnight at 4°C. Slides were washed in TBST and incubated with streptavidin conjugated Alexa-Fluor 594 (Life Technologies) for 2 hours at room temperature. Images were captured using the Zeiss LSM 700 confocal inverted microscope.
